# Multisensory Testing Framework for Advanced Driver Assistant Systems Supported by High-Quality 3D Simulation

**DOI:** 10.3390/s21248458

**Published:** 2021-12-18

**Authors:** Paweł Jabłoński, Joanna Iwaniec, Michał Jabłoński

**Affiliations:** 1 Department of Robotics and Mechatronics, Faculty of Mechanical Engineering and Robotics, AGH University of Science and Technology, Mickiewicz Alley 30, 30-059 Cracow, Poland; 2 Varroc Lighting Systems, s.r.o., Suvorovova 195, 742 42 Šenov u Nového Jičína, Czech Republic; Michal.ttl@gmail.com

**Keywords:** advanced driver assistance systems, object recognition, controller design and validation, hardware-in-the-loop, closed-loop testing, lidar simulation, CARLA simulator

## Abstract

ADAS and autonomous technologies in vehicles become more and more complex, which increases development time and expenses. This paper presents a new real-time ADAS multisensory validation system, which can speed up the development and implementation processes while lowering its cost. The proposed test system integrates a high-quality 3D CARLA simulator with a real-time-based automation platform. We present system experimental verifications on several types of sensors and testing system architectures. The first, open-loop experiment explains the real-time capabilities of the system based on the Mobileye 6 camera sensor detections. The second experiment runs a real-time closed-loop test of a lane-keeping algorithm (LKA) based on the Mobileye 6 line detection. The last experiment presents a simulation of Velodyne VLP-16 lidar, which runs a free space detection algorithm. Simulated lidar output is compared with the real lidar performance. We show that the platform generates reproducible results and allows closed-loop operation which, combined with a real-time collection of event information, promises good scalability toward complex ADAS or autonomous functionalities testing.

## 1. Introduction

Nowadays, passenger cars include electronic systems which help drivers on the road. This part of the automotive industry is growing rapidly and, in the near future, its goal is to deliver fully autonomous cars. The wide range of electronic components and sensors used to achieve this goal are called the Advanced Driver Assistant System (ADAS).

Naturally, large Original Equipment Manufacturers (OEMs) start to compete in the field of autonomous systems spending vast amounts of resources to develop systems capable of maintaining autonomy on any road and in any weather conditions. Each of these companies represents a different level of driving automation. Driving autonomy levels were defined by the Society of Automotive Engineers (SAE) in Standard J3016 [1]. Today’s most advanced vehicles support the third level of autonomy in the five-level SAE standard scale. The third level denotes partial automation, where the car is able to automatically steer, accelerate or break but the human driver still takes the responsibility for any eventual mistakes. The exponential increase in ADAS expertise, reliability and safety is required before the next level of automation can be achieved on public roads.

One of the challenges is to test and validate ADAS components on each level of development to meet all the customer requirements. Testing can consume a vast amount of resources and budget for projects. Those challenges are well-known in the automotive industry. There are many different approaches, which deal with these problems [2]. The V-Model [3] shows the approach of X-in-the-loop methods to test and validate any ADAS functionalities. Hardware-in-the-loop (HiL) is the system, which puts the hardware part of the ADAS under test. There are plenty of existing simulation frameworks built for ADAS system testing [4,5,6,7,8] that both share and are unique in various features and implementations compared to our work. Unlike our approach, some of the frameworks simulate the whole vehicle [7], others put a real vehicle onto the dynamometer’s bench to test the whole car in the loop [5,6]. Frequently, and similarly to our approach, a strong focus is placed on camera sensor testing in terms of lane-keeping assistance [8] or active cruise control. Additionally, our peers propose testing environments for components [9], sensors or even bigger system topologies [10,11] at the early stages of development.

The novelty of our proposal lies in the adoption of a real-time, hardware-in-the-loop simulation, otherwise commonly used for combustion or electric engine testing. With this strategy, we take advantage of a well-known, real-time technology for engine test benches and combine it with a state-of-the-art 3D rendering simulation. Moreover, we demonstrate that such a validation system can be conveniently run on a single, standard PC where a Windows operating system and a real-time subsystem interact over a shared memory space.

There are a few types of already known HiL systems, such as Non-Performance HiL (NP-HiL), Open-Loop HiL (OL-HiL) and Closed-Loop HiL (CL-HiL). NP-HiL is used to validate the integration of the system. It verifies the communication between system components but is not able to perform any functional tests on ADAS hardware. OL-HiL uses already existing data or logs to inject into the hardware and validate the ADAS functions and performance. CL-HiL not only validates the system output but also works in a feedback loop with hardware and can react to the simulated conditions. This type of HiL system is used, e.g., to validate automatic braking or lane-keeping functions without going on the road. Engineers face many technical and conceptual barriers during the development of HiL test benches [9,12] as the complexity of car systems increases rapidly to fit into autonomous driving function requirements [13]. Among some open topics are how to simulate multiple sensors including radar, ultrasonic, video or lidar in OL or CL-HIL. The HiL system proposed in this paper attempts to combine validation abilities of non-functional and functional test benches into one, flexible and high-performance HiL, without any drop in performance or test quality.

To achieve these benefits the HiL has to meet some basic requirements. The first requirement is to validate the hardware component of the vehicle ADAS system, which we will refer to as the Test Object (TO) throughout the rest of the article. A HiL system should prove its deterministic properties in time performance and generate repeatable results. The validation process should be focused on real-time [14] measurement capabilities supported by a real-time operating system. The software parts of the system have to be compatible with Microsoft Windows programs broadly used by OEMs. A flexible and user-friendly bench environment should support operators from various customers. One way to achieve this purpose is to build a system with device-oriented structure planning from scratch. It means that the environment should be able to easily support new devices and applications necessary in order to meet the specific expectations of different customers.

To prove the performance and capabilities of the proposed system a few experiments are performed. First, an open loop, traffic sign detection problem is used to determine the real-time measurement capabilities of the system. Second, a lane-keeping algorithm validation in a closed-loop setup, with a real-time road rendering scenario is run. The third experiment provides Velodyne VLP-16 lidar sensor simulation, which is used for free-space detection in front of the vehicle. The data from a simulated lidar are compared with real sensor data.

## 2. Architecture of the Proposed System

The main goal is to set up a measurement system that provides stable and reliable results. Literature and experimental investigation lead us to the use of the Morphee software platform [15], which covers automation, ECU (Electronic Control Unit) calibration, real-time simulation and measurements. This platform is able to operate on the real-time kernel provided by Interval Zero RTX, more precisely described in chapter five. Morphee provides up to one hundred parallel task handling and thousands of measurement channels. This software is broadly used around the world for combustion or electric engine test benches. Morphee abilities were used to build the ADAS test bench. All the experiments described in this paper are run, controlled and automated by Morphee, which is the core of the validation system.

**Figure 1:**
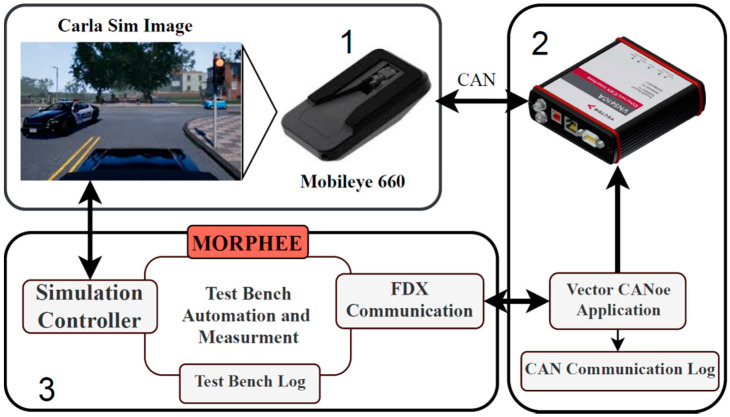
Diagram showing the three most important elements of the proposed HiL system. 1—Hardware part of framework, sensor and screen, 2—Can communication block VN5110A plus harness and Canoe, 3—Morphee automation software block.

**Figure 2:**
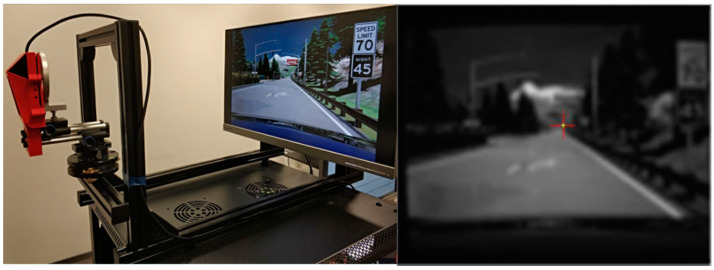
Left: HiL frame with Mobileye and display attached. Right: low-quality image captured for calibration purposes.

The following block diagram (Figure 1) explains the proposed open-loop HiL architecture prepared for the first experiment. In this test, the TO is the Mobileye 660 camera commonly used in research as a state-of-the-art, automotive sensor [5,6,16,17]. Mobileye 6 integrates a few ADAS functionalities such as traffic sign recognition, lane-keeping and emergency braking. Figure 1, block 1 physical configuration is further shown in Figure 2. The aluminum frame holds a display monitor, mounted in front of the TO. The TO is also attached to the same frame in a precisely calibrated position with respect to the display. The image on the display is generated by CARLA simulator v.0.9.9, which is controlled by the Morphee platform (Figure 1, block 3). Simulation of a vehicle bus (Figure 1, block 2) is handled by the CANoe application [18]. Together with the dedicated hardware (VN5610A), it transmits data between the TO and Morphee. A simulated bus looks exactly like a car but without the necessity of using any real vehicle ECU. For the purpose of communication between TO and Morphee, the CANoe and Fast Data Exchange protocol (FDX) are used [19]. This protocol is specially designed for Canoe application to allow an external application (here Morphee) to control the simulated CAN bus.

Morphee (Figure 1, block 3) manages the data flow and control process between each of the parts. In summary, block one is used to generate input data for the TO. In the case of the camera, it is the live generation of the image. Part two is responsible for handling the communication and control of the TO. In the case of Mobileye, it is a CAN bus with an open protocol. (Open protocol means that the camera sends out all the information from detections and real-time calculations on the CAN bus). The last part of the job is to synchronize and properly log all measurements and data with a high precision timestamp into the result file. The most important issue for this task is to provide a real-time-based clock for the purpose of logging and synchronization. This can be carried out with Morphee thanks to the fact that it runs on the RTX kernel.

## 3. Image Generation and Projection

For the purpose of simulating a road scenario, the camera is positioned carefully on a fixture relative to the monitor in order to reproduce a field of view similar to a real car application (Figure 2, left). There are a few ways to capture the road image by the sensor. The easiest way is to play a video recorded on the road in front of the camera. This simple solution with a high-quality display and with a refresh rate at least twice the camera frame rate is used. For Mobileye 660, a display with a 60 Hz refresh rate is sufficient. It is important to avoid any light reflections on the display which could cause incorrect detections.

The monitor aspect ratio can be different than that of the camera sensor, therefore the maximum distance between the camera and monitor was calculated to ensure that only the displayed image fills the camera field of view. In the next step, the TO is calibrated using a camera manufacturer software-generated road image (Figure 2, right).

The actual TO test is a simple, open-loop configuration (OL-HiL) where Morphee uses a video file recording of a CARLA simulation displayed on the computer screen. Mobileye 660 with an open CAN bus allows us to see whether the TO detects objects such as cars, pedestrians, lines or traffic signs during the video playback.

However, little stands in the way for more sophisticated testing in the closed-loop since Morphee can control the CARLA simulator to generate real-time images as well. This allows the HIL system to put ADAS functionalities such as a lane-keeping assistant or automatic breaking under various tests representing potentially rare and otherwise dangerous road conditions and events in reliable, repeatable and foremost, safe, simulated conditions. In this paper, both types of tests: open- and closed-loop are performed and described.

## 4. CAN Bus Simulation

**Figure 3:**
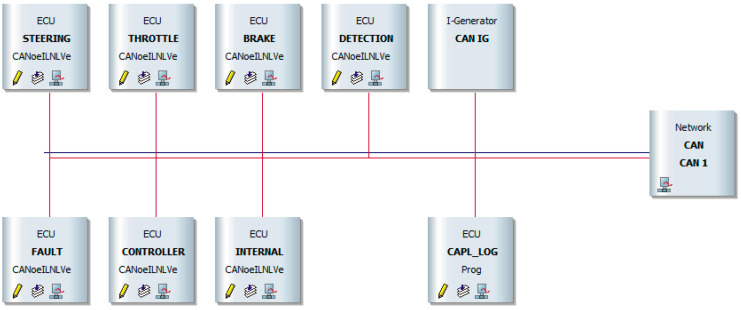
Setup of communication created in CANoe which mimics vehicle CAN bus.

Mobileye camera is an aftermarket sensor with ADAS functionalities designed to work in cars that do not have such a system built-in. The sensor has to be connected to the car CAN bus to operate. In order to work correctly, the camera requires vehicle data such as speed, the state of turn indicators, brake lights, high beam, etc. The same communication data have to be sent to the sensor in case of HiL tests. For this purpose, we used Vector CANoe. Equipped with specialized hardware, Vector CANoe allows to configure and simulate a car’s CAN bus that reproduces the communication environment for the TO. Figure 3 shows an example schematic vehicle bus with all nodes required for the TO operation.

Communication nodes (visible in Figure 3) mimic vehicle ECUs, connected to the bus together with the “Network” node. The “Network” node represents the external hardware box where the TO is connected to the simulated bus. The I-Generator node simulates CAN messages that are sent out from the vehicle to the TO, which are required for the correct functioning of the TO. We use a Mobileye sensor with enabled CAN debug so that the sensor not only indicates warnings on its display but also broadcasts detection and measurement data. For this purpose, the second CAN bus is defined. This second bus uses another hardware channel of CAN in the Vector box and allows the HiL system to read the data computed by the sensor. Both of the CAN communications are available in the Morphee platform via the FDX protocol (Figure 1, block 2). This protocol allows Morphee to control CANoe measurements and read and write necessary data into the CAN bus. For example, Morphee sends simulated vehicle speed into the sensor, which the TO uses to calculate time to collision. In turn, time to collision can be read by Morphee via the same communication protocol. It is worth mentioning that the FDX protocol driver at the Morphee side is supported at the level of the RTX subsystem, described in detail in the next chapter.

## 5. Automation System

Research into existing automation systems that can work as an HiL environment platform covered applications such as Windows Subsystem for Linux (WSL), LabView with Python integrator toolkit, Matlab Simulink with ROS toolbox, ViCanDo or Robotic Operating System. Each of these has some advantages and disadvantages. However, below, we argue that Morphee fits the HiL project requirements the best.

**Figure 4:**
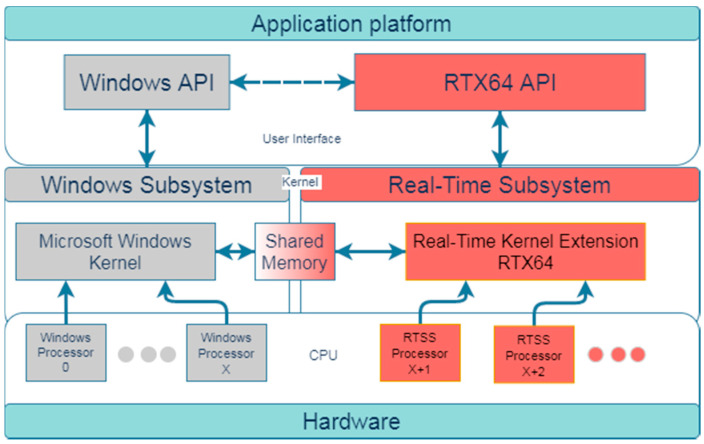
Interval Zero RTX64 subsystem general diagram. A more precise diagram is provided on the Interval Zero website [21].

The real-time performance of Morphee is based on its ability to run processes on the RTX subsystem [20]. The diagram in Figure 4 explains how the RTX subsystem uses separate hardware parts to run and control its own processes.

RTX64 extends but does not modify the Windows hardware abstraction layer (HAL) by adding a real-time subsystem (RTSS) to Windows that delivers deterministic, real-time performance. Morphee is able to run its processes on the RTX subsystem, which enables real-time measurement and communication processes. This is performed via allocating part of the CPU cores only for the RTX subsystem’s usage. In the case of the proposed HIL system, two out of twelve cores of a computer’s CPU are allocated for the RTX subsystem’s exclusive usage. Installation of RTSS turns standard Windows-based systems into hard, real-time machines, without the necessity of expensive, specialized hardware. Moreover, the final configuration still allows to run standard Windows-based applications and share data between Windows and the RTX subsystem. A similar approach, with the usage of real-time code generated from Matlab/Simulink Embedded Coder Toolbox is used in [6]. The important difference, however, is that this research relies on the use of a dedicated external DSpace MicroAutobox-II hardware to run the simulation system in real-time mode, while our system integrates the real-time support on the same computer.

## 6. Real-Time Measurement of Detection Events

### 6.1. Experiment Description

The proposed HiL environment must provide reliable and repeatable results. Accurate measurements in terms of time are crucial for any validation environment. To provide correct results all gathered data have to be read and validated or stored in real-time. An experiment was set up based on the comparison of time calculated by two separate clocks. The first one was a reference, the FPGA clock (Vector VN5610A) while the second one was for the clock to be verified—the Morphee kernel clock—which has its source in one of the CPU cores. This external FPGA clock serves as a reference for Morphee environment measurements.

**Figure 5:**
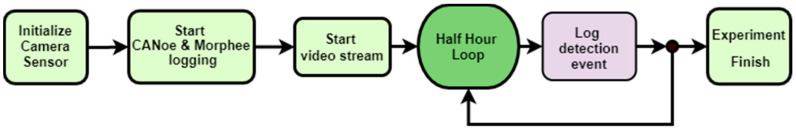
Flow chart of test for the experimental verification of measurement capabilities of the HiL system.

A properly implemented HiL system should provide a measurement accuracy at the level of Morphee clock sampling time. The highest sampling rate supported by the Morphee system is 1 kHz. Figure 5 depicts the flow chart of the performed test.

**Figure 6:**
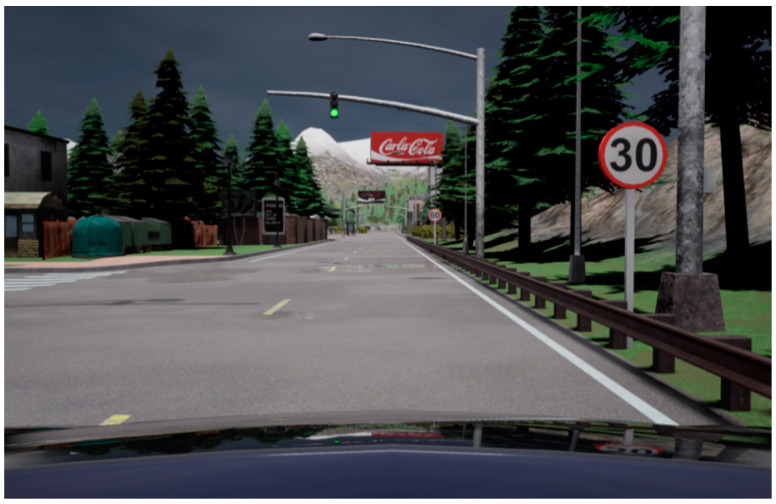
Sample image from video used in experiment with two speed limit signs visible.

The first step of the test consists of the camera initialization. Initialization starts the power supply for the Mobileye sensor and CAN bus simulation hardware. The next step triggers the logging process in CANoe and Morphee. Both CANoe and Morphee logs are set up to capture detections of traffic signs sent out by the sensor over the CAN bus. Each log file contains its own timestamp, one is calculated by Morphee and an independent one is from the FPGA clock. The third step starts the video with a road scenario. The scenario included a straight road with two speed limit signs standing on the right side of the road. A frame with visible signs is shown in Figure 6.

The video is looped so the sensor should detect the signs for the duration of the experiment. Thirty minutes were set for one experimental verification of the system. The CANoe log has its time source in the hardware FPGA clock, and it is used as a reference to compare with the Morphee system measurements. The next subsection describes the analysis and results gathered in this experiment.

### 6.2. Experiment Results

**Figure 7:**
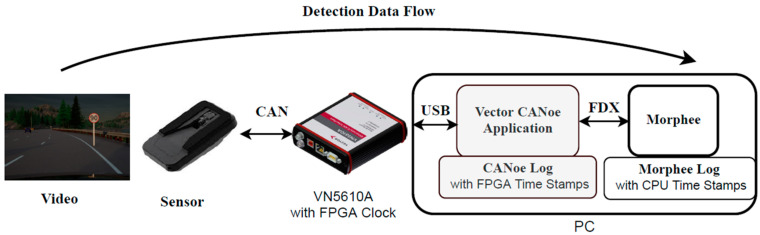
Sign detection data flow diagram.

Detection data flow is depicted in Figure 7. CANoe data are triggered by the Morphee environment and this trigger time is the first information saved by the experiment. It is worth stressing that Mobileye sign detection functionality [22] does not send cycling messages with detected sign information. Sign detection is event-based, therefore it is only when the sensor detects a sign that it sends a CAN frame with all the information about it. The message includes information about sign position relative to the car, the sign classification and if it contains some supplementary signs.

The key point is that, unlike the FPGA clock timestamps, the Morphee log stores the sign detection events past the FDX protocol. Meantime FDX communication is cyclical and initialized by Morphee to exchange data every millisecond. Log sampling in Morphee is also set to 1 ms. FDX communication sends a request for data every millisecond. Each data request has to be received by CANoe which reacts and sends a response with asked data. Data send and receive over FDX communication should not cause more than 2 ms of error (max 1 ms for send and max 1 ms to receive data). Additionally, Morphee log sampling should not cause more than 1 ms of error. Summing up maximum measured error should not exceed 3 ms.

**Figure 8:**
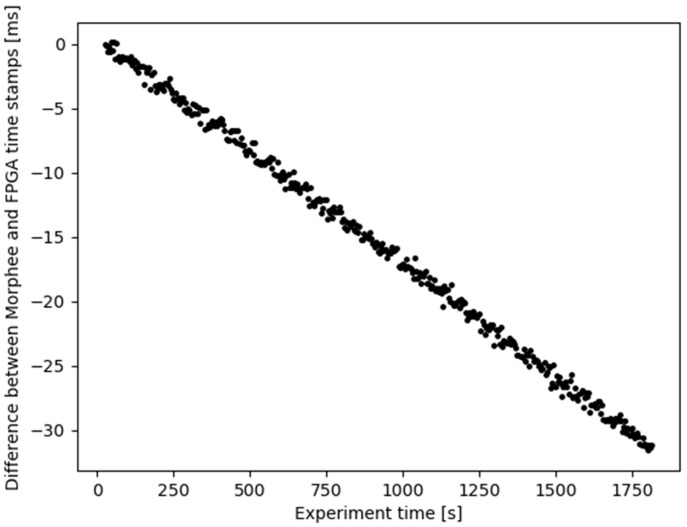
Morphee timestamps relative to FPGA time. Each dot represents one sign detection event.

For better visualization of logged data, further graphs normalize data as a delta to reference FPGA clock timestamps. The graph in Figure 8 illustrates Morphee logged data gathered by the system relative to the reference FPGA clock.

**Figure 9:**
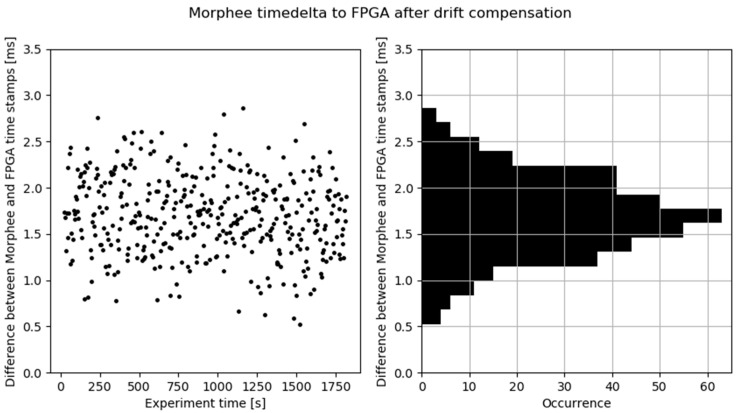
Time delta between Morphee and FPGA clock after time drift compensation.

The first detection event stored in the log was used to synchronize Morphee and FPGA time. The full experiment captured 408 events. Figure 8 shows an unexpected result, the Morphee stamps are ahead of FPGA clock time. This situation should be the opposite, considering that data has to be sent over FDX communication after detection and then logged by the Morphee environment. The reason for such a situation is the time drift, which exists between the reference and the Morphee clock. The difference starts from zero due to the synchronization and accumulates continuously throughout the experiment reaching around 31 milliseconds at the end of the test loop. The time drift calculated out of these data is 1.056 ms per minute. Figure 9 depicts the same experimental results but after compensating for the clock drift.

The goal of this experiment was to prove that Morphee measures do not exceed the expected maximum error of 3 ms. This is indeed confirmed by the system performance data presented in Figure 9, where none of the events measured by the Morphee system exceed the expected maximum time difference against the reference clock. In most of the referenced literature [4,5,6,7,8], which performs ADAS HiL validation, we do not find mentions about system performance and measurement errors. In [6] it is stated that the HiL oscillation for the lane-keeping algorithm might occur due to structural delays of the HiL. In [4], a 100 Hz system operational frequency is presented. By comparison, the presented Morphee HiL platform allows 10 times faster sampling.

## 7. Mobileye 6 Simple Lane-Keeping Algorithm Run in Closed Loop

### 7.1. Camera Line Detection

The Mobileye 6 sensor supports line detection. If a vehicle starts to cross one of the lane boundaries the system is expected to generate a warning sound. Lane markers are detected and described by the system as a third-degree polynomial model (1). The model is expressed as function *X*(*z*), where *z* is the longitudinal distance from the sensor and *X* is the lateral distance from the sensor:

(1) $$X\left(z\right)=6C_{c}\cdot z^{3}+2C\cdot z^{2}+\tan\left(H\cdot z\right)+C_{0},$$

where $C_{c}$—curvature rate [1/$m^{2}$], C—curvature [1/m], *H*—heading angle [radian], $C_{0}$—distance to line for *z* = 0 [m].

All four polynomial parameters are readily calculated by the sensor firmware and broadcast over the open CAN communication protocol. Additionally, the sensor provides a view range value. View range informs how far the sensor sees the lines. This parameter is calculated only while vehicle speed is greater than zero.

### 7.2. LKA—Lane Keeping Algorithm

Mobileye sensor line detection was used as the input for a simple lane-keeping algorithm. The sole purpose was to be able to run a closed-loop scenario on the HiL system. For this purpose, we found it sufficient to approximate the inputs to the second-degree polynomial. Based on the curve parameters, LKA calculates the destination point in front of the vehicle and subsequently, the appropriate steering wheel angle. Formula (2a–d) define how the destination point is calculated out of the input parameters, while Figure 9 illustrates the resulting geometry:

(2a) $$L_{c}=\frac{\left(\;\left|C_{0l}\right|+\left|C_{0r}\right|\;\right)}{2}\;,$$

(2b) $$X_{l}\left(z\right)=2C\cdot z^{2}+\tan\left(H\cdot z\right)+C_{0}-L_{c}\;,$$

(2c) $$X_{r}\left(z\right)=2C\cdot z^{2}+\tan\left(H\cdot z\right)+C_{0}+L_{c}\;,$$

(2d) $$Z_{d}=\frac{\left(X_{l}+X_{r}\right)}{2}\;,$$

where $C_{0l},C_{0r}$= $C_{0}$ curvature parameters (1) for left and right line [m], $L_{c}$ = middle of lane for *z* = 0 m, $X_{l}$, $X_{r}$ = destination point lateral distance for left and right line [m].

For the purpose of the HiL experiment, distance *z* for destination point was chosen as three-quarters of the view range. The destination point is further used by the algorithm to calculate the radius of the curve for the vehicle to follow according to a circle Equation (3):

(3) $$Ax^{2}+Ay^{2}+Bx+Cy+D=0$$

**Figure 10:**
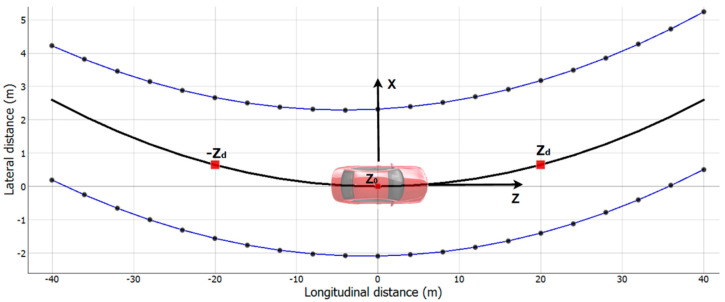
Example lane markers model with destination point $\;Z_{d}$. The black center line is the curve for the vehicle to follow.

Destination point $Z_{d}$, with its reflection point relative to the *X*-axis –$Z_{d}$, and point 0,0 are used to define the circle and therefore obtain the radius (Figure 10). The following Formulas (4) define how to calculate the radius of the circle that these three points belong to:

(4a) $$A=x_{2}y_{3}-\;x_{3}y_{2},\;$$

(4b) $$B=\left(-y_{3}\right)\left(x_{2}{}^{2}+y_{2}{}^{2}\right)+y_{2}\left(x_{3}{}^{2}+y_{3}{}^{2}\right),$$

(4c) $$C=\left(-x_{2}\right)\left(x_{3}{}^{2}+y_{3}{}^{2}\right)+x_{3}\left(x_{2}{}^{2}+y_{2}{}^{2}\right),$$

(4d) $$r=\sqrt{\frac{B^{2}+C^{2}}{4A^{2}}}\;\;,$$

where $(x_{1},y_{1})=\left(0,0\right),\;Z_{d}=\left(x_{2},y_{2}\right),-Z_{d}=x_{3},y_{3\;,\;}$, r—curve radius.

Following the lane curvature radius r we can define the vehicle wheel turn angle γ using Equation (5).

(5) $$\gamma =\tan^{-1}\left(\frac{L}{r}\right),$$

where: L—distance between vehicle axles [m].

In our evaluation, we used the CARLA simulator Tesla model 3 with an axle length of approximately 3 m. The last step for the algorithm is to translate the wheel angle into the steering wheel angle. This function was defined empirically in simulation by measurement of steering wheel position and wheel angle. The relationship was found to be linear with a slope of 0.03. The algorithm does not use vehicle speed as an input parameter.

### 7.3. Real-Time Closed-Loop Run of Lane-Keeping Algorithm

**Figure 11:**
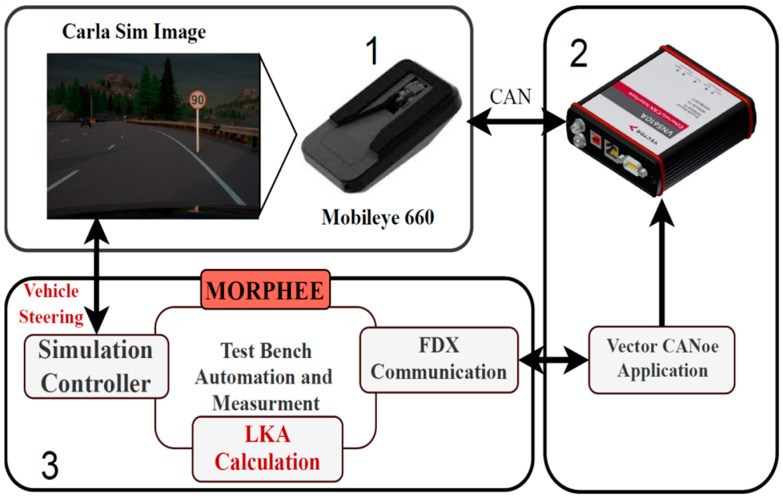
Block diagram of lane-keeping algorithm test run on HiL closed-loop setup. 1—Hardware part of framework, sensor and screen, 2—Can communication block VN5110A plus harness and Canoe, 3—Morphee automation software block with LKA algorithm implemented.

Closed-loop testing requires the creation of a feedback loop based on the input generated by the sensor. In the case of LKA, the steering input generated by the algorithm must change accordingly to the image generated by the simulation. Schematic data flow are shown in Figure 11.

A simulated vehicle model starts to drive straight at a constant speed. The camera sensor turns the generated image into CAN bus data. Via FDX protocol, these data are received by Morphee environment where LKA estimates the steering wheel angle. Computed data are set into simulated vehicle steering in real-time. This input creates an instant change in the vehicle position on the lane. Line position is again detected by the sensor and the data loop is closed. Mobileye sends new detection data every 60 ms. Additionally, vehicle data such as longitudinal speed, are sent back via Morphee and FDX into the sensor CAN bus. This experimental setup was tested on one of the CARLA simulator multi-lane highway tracks, shaped into a loop.

**Figure 12:**
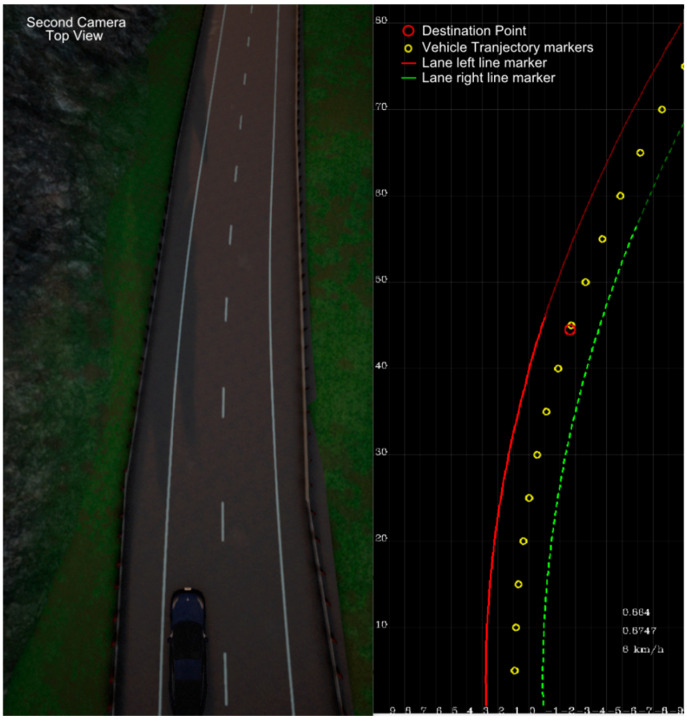
Left: camera view above the car for comparison with line detection data. Right: visualization of line detection (red-lane left, green-lane right) with destination point and trajectory markers obtained from Mobileye camera view as in Figure 13.

**Figure 13:**
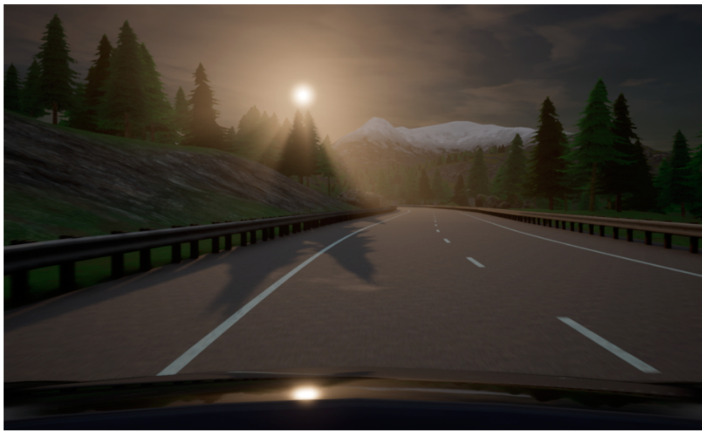
Mobileye 6 view of a curve subject to LKA.

For the purpose of the experiment and measurements, the work of LKA is drawn in an additional window which allows for comparing the CARLA top road view (Figure 12 left) with the result of line detection (Figure 12, right). The LKA window includes both road lines detections (red and green) and the estimated vehicle trajectory (yellow dots), based on the destination point. This is the trajectory for the vehicle to follow. Additionally, each line model indicates a valid range by color intensity change. The red circle is the destination point estimated from the lane model. Figure 13 represents the Mobileye view of the same scene.

The simulation road curve can be compared with the detected lane through the second camera top view. Such a simple lane-keeping algorithm implemented in the HiL system is able to drive on an endless loop continuously without any steering interventions up to a speed of about 50 km/h. If the vehicle speed exceeds 50–60 km/h the algorithm starts to fall into oscillations and misses sharper turns. Once the vehicle camera losses sight of the lines, the vehicle crashes into the road boundary. Most probably the speed limitation occurs due to the simplified lane model which was used to define lane marking. The third-degree polynomial, which uses curvature change parameters for lane shape estimation, could be better as an input for the algorithm. Additionally, Mobileye lane marker parameters detection sampling of 60 ms can be too slow in the case of higher vehicle speed. That being said, the purpose of the algorithm was not to provide perfect lane-keeping functionality but to allow for the complete closed-loop setup to be placed under a test in a safe and reproducible manner. A similar experiment for the lane-keeping assistant test is performed in [6], except that the whole vehicle with a Mobileye sensor is put under test. 

## 8. Road Free Space Detection on Velodyne VLP-16 Lidar Point Cloud

### 8.1. Lidar Sensor Velodyne VLP-16

Lidar sensors are already commonly used in autonomous system prototyping and development. Lidar provides precise information about vehicle surroundings, especially in terms of distance to objects. The sensors are currently relatively expensive and, therefore, mostly used as ground-truth for radar or radar-camera systems. Nevertheless, lidar technology is becoming cheaper each year and starts to be used more commonly in high-volume projects. For this reason, the simulation capability of a lidar system is also of high interest to support future autonomous systems’ validation.

**Figure 14:**
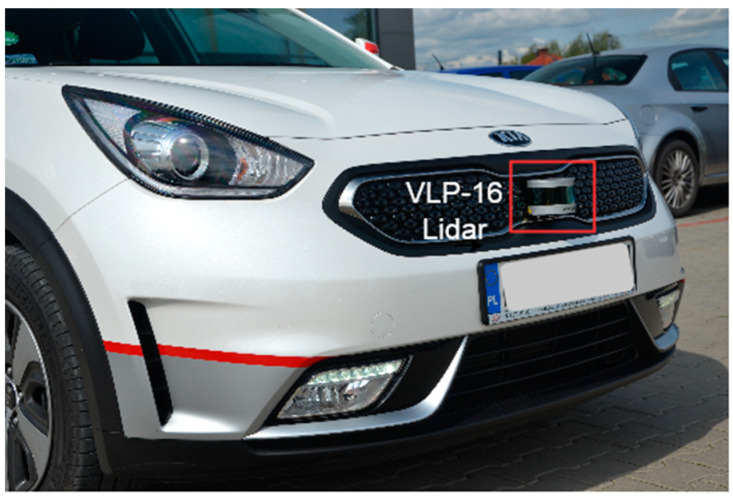
Velodyne VLP-16 lidar mounted at the front of a test vehicle. The vehicle is equipped with Nvidia AGX development system which analyzes data from the sensors.

Velodyne VLP-16 is one of the popular lidar sensors used in many research projects [23,24,25]. This sixteen-channel lidar sends data via Ethernet user datagram protocol (UDP). Smart Vehicle research project of FEV Company [26] is one of the examples where VLP-16 lidar is used to develop ADAS functionalities. Mounted on a real car (Figure 14), the lidar generates data that can determine what part of the road in front of the car is occupied or not using what we call a free space detection algorithm.

We developed the HiL system to support lidar sensor simulation in conjunction with the 3D CARLA simulator. The output of the lidar simulation is a 3D point cloud and each point is a reflection out of the simulated world object. Lidar simulation input, reshaped in the Morphee environment, allows for mimicking the Velodyne VLP-16 output. This HiL feature allows us to pre-validate the free space detection algorithm without the necessity to drive a real vehicle.

### 8.2. Simulation of VLP-16 Lidar

The lidar emits a short light pulse, which reflects from surfaces and is received back by the sensor. The distance to any object can be estimated based on the Time of Flight (ToF) of the light. Because ToF is very short, it is difficult to produce any kind of simulation environment for lidar hardware. However, as long as the hardware does not have to be tested, only data produced by the sensor are sufficient for downstream system/algorithm analysis. Consequently, the simulation provided in this chapter mimics only the lidar data output.

Lidar simulation starts from setting up lidar model parameters in the CARLA simulator. Table 1 lists the parameters of simulated lidar with comparison to the physical VLP-16 lidar manufacturer parameters.

Most of the real lidar parameters can be set directly in the simulation model but not all. In the case of vertical angular resolution, its value results from the vertical field of view (FOV) and the number of channels. Lidar has 16 channels and a vertical FOV of 30 degrees. To fit into all channels vertical resolution must be ~2 degrees. Estimation of horizontal resolution is a little more complicated and requires the sampling rate. Following Equations (6) and (7) define how points per second $P_{ps}$ value is calculated out of other lidar parameters.

(6) $$P_{ps}=\frac{FOE_{H}*N*f}{\varphi_{H}},$$

where—$FOE_{H}$ horizontal field of view [°], N—number of channels, f—rotation speed [Hz], $\varphi_{H}\;$ = horizontal angular resolution [°].

(7) $$P_{ps}=\frac{360{}^{\circ}*16*10\;\mathrm{Hz}}{0.4{}^{\circ}}=144000\;\frac{1}{s},$$

**Figure 15:**
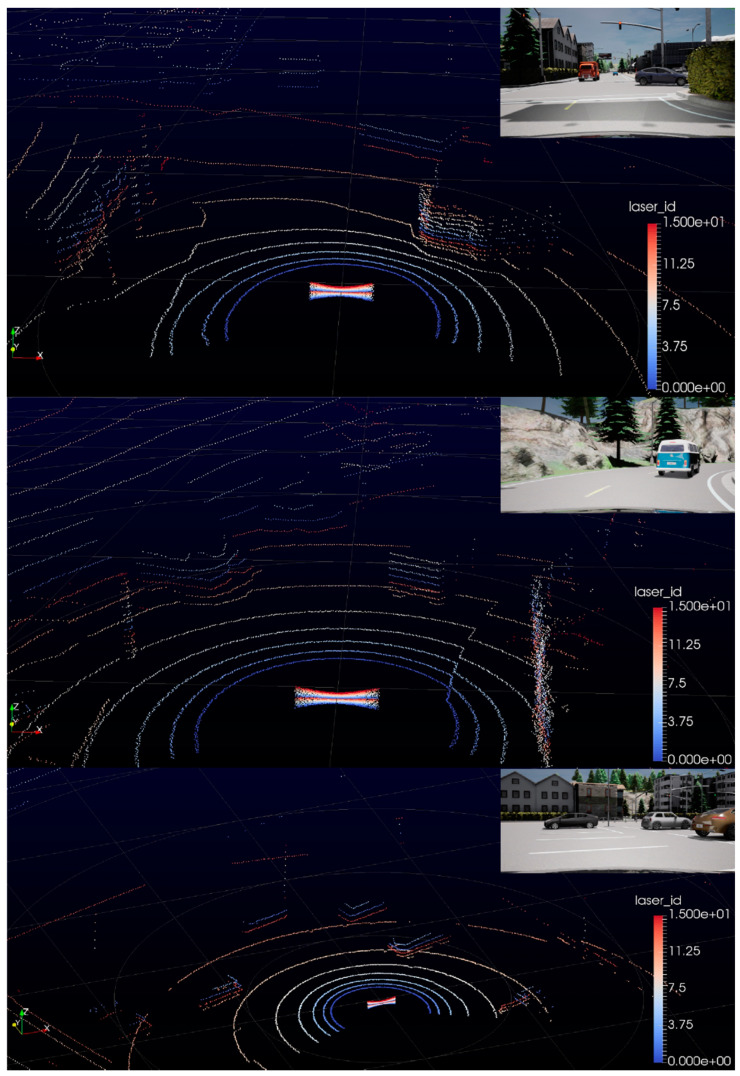
Velodyne VLP-16 lidar simulation visualized in manufacturer software tool VeloView.

The sensor is positioned at the front bumper of the simulated vehicle, the same place as on the real vehicle. Morphee receives point cloud from CARLA as generated by the simulated lidar. Each point is described by a set of Cartesian coordinates *x*, *y*, *z* expressed in meters. Point cloud has to be reshaped into Velodyne lidar data shape. Velodyne sends out each point described as distance and azimuth angle within the specified channel (horizontal angle). That is why the Cartesian points are converted to a cylindrical coordinate system as a first step. Then, each point is assigned to the correct channel. In this place, the Morphee system considers real sensor range precision. Range precision informs about lidar distance measurement error. The CALRA-simulated sensor measures the distance to objects in an idealized way, resulting in the simulated points cloud being unrealistically perfect. That is why to each obtained data point a random value from ±3 cm range and a uniform distribution was added in order to mimic the precision of the real sensor. Reshaped data forms byte stream into the HiL Ethernet card. The card is set to work with the same IP address as the real hardware lidar. Morphee output for the VLP-16 lidar simulation is a byte stream over UDP (Figure 15).

Velodyne lidar simulation is verified on VeloView software which is the lidar manufacturer’s own data cloud visualization tool. We input the simulated data into the VeloView to confirm the basic functionality of the simulated lidar against specific CARLA scenes. Simulation mimics communication protocol and sensor point cloud. A few examples from the VeloView tool are shown in Figure 15. Examples also include a camera view from the same point in time.

Simulation works as expected, moreover, the point cloud is rendered in real-time by VeloView. In the images, it is visible how the distance of each point is randomized resulting in slight noisiness.

### 8.3. VLP-16 Lidar Free Space Detection Experiment Result

**Figure 16:**
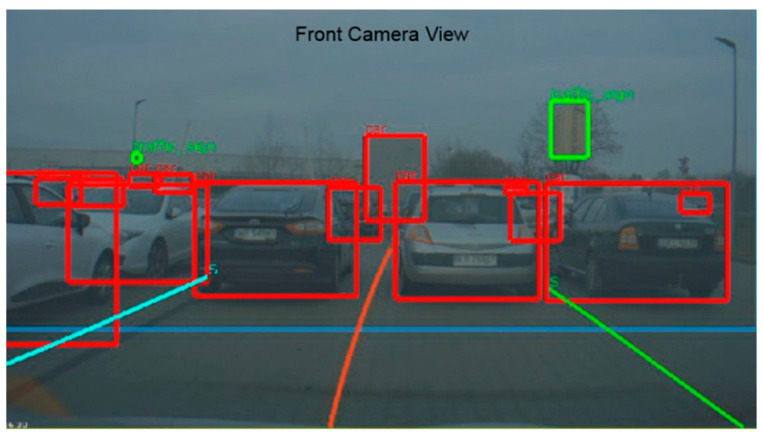
Car front camera view from the same point in time as in Figure 17.

**Figure 17:**
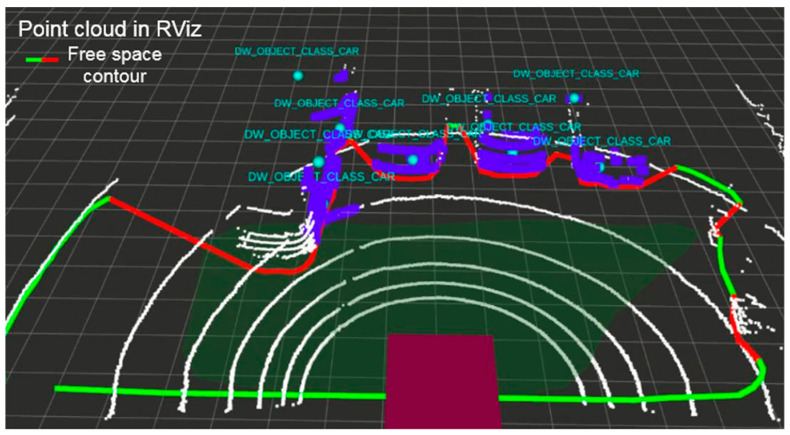
VLP-16 lidar point cloud visualization with contour of detected free space in front of the vehicle.

To compare the simulated and real system performance let us first take a look at the physical free space detection system architecture. The Nvidia AGX board installed in the vehicle trunk runs a Robotic Operating System (ROS) on its Ubuntu system. ROS helps to read input data from many different sensors, visualize them and perform computations. The lidar input point cloud is computed by AGX into the free space detection in front of the vehicle. Visualization of point cloud and detection is performed in Rviz, a 3D visualization tool of ROS. Figure 16 shows an example road scenario and Figure 17 is the corresponding lidar data overlaid with the result of the interpretation algorithm implemented and run in the AGX system.

The lidar point cloud is drawn in form of white points (the points are large, therefore they may give the impression of lines in Figure 17). Most important are the green contour and the red line that correspond to the free and occupied space in front of the car. Figure 16 illustrates a parking scene, where four cars are parked in the lidar view. On the basis of the lidar data, the algorithm is, therefore, able to define what part of space in front is drivable. Each object which is higher than a few centimeters is treated as an obstacle, which should not be hit or crossed by the vehicle.

**Figure 18:**
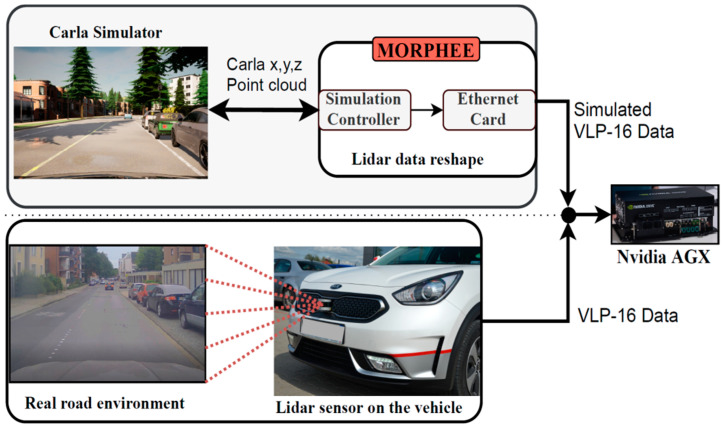
Data flow diagram of VLP-16 lidar simulation for free space detection algorithm and standard flow of data for real hardware lidar in vehicle.

Following this, we wish to verify the feasibility of free space detection on simulated lidar data. To that end, we utilized the very same Nvidia AGX hardware. Figure 18 provides a scheme, which explains how the simulation replaces a real sensor data input into the AGX system.

**Figure 19:**
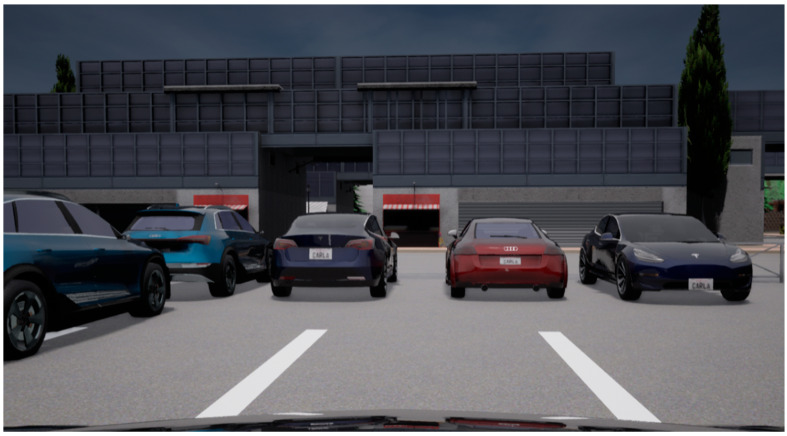
CARLA parking scenario forward view.

To qualitatively compare the physical and simulated result a similar situation as in Figure 16 is created in CARLA using a parking lot space (Figure 19).

**Figure 20:**
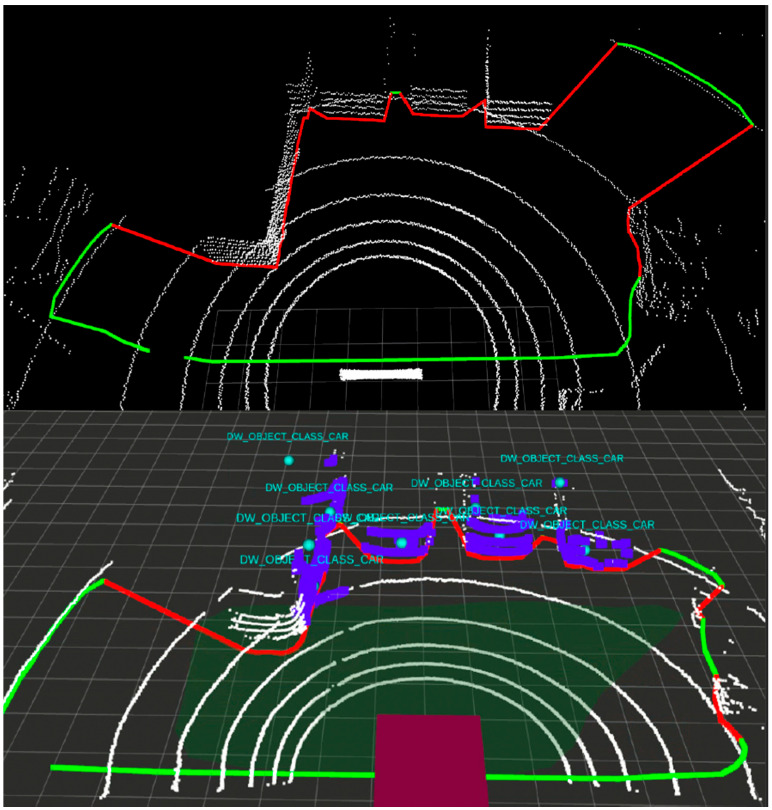
Comparison of Velodyne lidar with free space detection algorithm run on real and simulated data.

Using this scenery, the Velodyne lidar simulation is run and sensor data are injected into the Nvidia board to compute and visualize if the free space detection algorithm works as intended. Figure 20 compares the result of the free space detection algorithm based on the simulated and physical lidar data. On the simulated point cloud (Figure 20 top), the free space contour is drawn similar to the real data (Figure 20 bottom). The free space algorithm correctly defines what part of the road surface in front of the vehicle is occupied, and which is not. All four visible parked cars are marked with a red line.

CARLA vehicle or obstacle models are not as highly detailed as the real data. This is clearly visible on the lidar point cloud. Most of the vehicles have a boxy-shaped collision area— certainly an item for future improvement.

What is difficult to demonstrate on a single image as in Figure 20 is that the developed system is capable not only of generating but also of converting and injecting data in real-time. The biggest difference between real and simulated results is the shapes of vehicle models, but this disadvantage can be improved upon with the usage of a newer version of the CARLA simulator.

## 9. Discussion

The real-time capabilities of the system were experimentally verified in the open-loop camera-based test. Experiment results were compared with an external, high precision time source generated by an FPGA microprocessor confirming that Morphee operates in real-time. It is noteworthy that in comparison to referred projects, our HiL system performance is equal, or even in some cases higher, without the necessity to use additional, dedicated hardware [4,5,6,7,8].

To showcase closed-loop test bench capability we implemented a basic lane-keeping functionality. The experiment showed that, up to a certain speed, the closed-loop setup with a camera sensor is capable to continuously control the simulated vehicle to keep it within its lane giving the possibility to run useful closed-loop validation experiments.

Moreover, Velodyne VLP-16 lidar sensor simulation was run and compared to real lidar data. Based on simulated lidar data, the free space detection algorithm was verified. The algorithm fed with simulated point cloud correctly detected free and occupied spaces in front of the simulated vehicle, similarly as it does for real sensor data. At the same time, CARLA sensor models are fully configurable, therefore the system is not limited to the specific hardware simulation models we have showcased.

## 10. Conclusions

In this paper, a hardware-in-the-loop automation system was proposed and experimentally verified. The system combines the following features:

- an innovative 3D simulation software—CARLA;
- utilizes a well-known, Morphee environment for automation and measurement control;
- has real-time capabilities based on Morphee processes running on RTX subsystem;
- does not require the use of dedicated hardware to perform real-time validation;
- allows closed and open HIL testing scenarios;
- allows to relatively easily add multiple sensor models; and
- offers a friendly, Windows-based, user interface.

**Figure 21:**
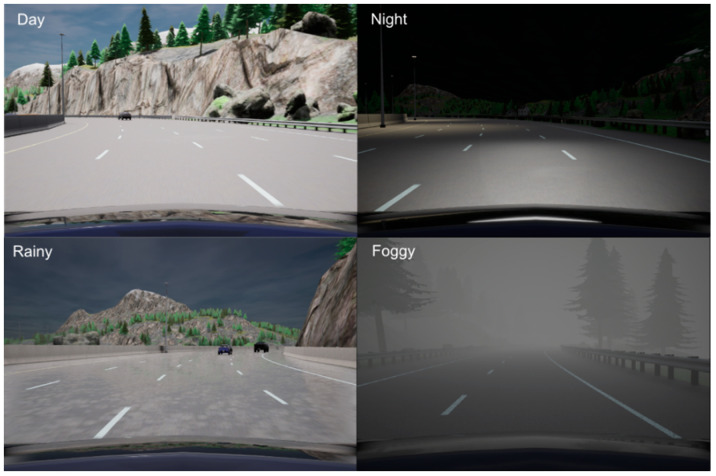
Example of four different weather conditions in CARLA simulator. Top left: clear daytime, top right: night drive, bottom left: overcast/rain, bottom right: foggy conditions.

We claim that CARLA is an excellent choice for the test bench purpose due to its advanced graphical engine (Unreal Engine 4). Integration of Morphee and CARLA into one hardware-in-the-loop automated system allows for performing testing with complex road scenarios. This gives the promise of Lidar or camera simulations that will speed up the development process of ADAS algorithms at a lower cost, without the necessity to drive the car on a road or a testing track. In general, CARLA’s capability to generate multiple views of the simulator at the same time comes in very handy, not only applied to cameras but also to a variety of different sensors available in the simulator. It is finally noteworthy that CARLA supports various weather condition simulations. This feature can be beneficial for driver assistance system testing. Figure 21 depicts examples of different weather conditions rendered by the simulator.

The HIL system is in constant development. The new version of the CARLA simulator gives new possibilities and new sensor models. Increasingly more complex architectures can be validated to speed up the early development process and aid the end system architecture decisions. The high integration level of the system allows for considering the possibility of sensor fusion for even more complex autonomous functions testing, with the only hard limitation being the performance of the PC workstation which must run both the simulations and Morphee.

## Figures and Tables

**Table 1:** Simulated and manufacturer VLP-16 lidar parameters.

Parameter	Lidar Simulation	VLP-16
Number of Channels	16	16
Measurement range [m]	100	100
Range Precision [cm]	-	±3
Vertical field of view [°]	+15/−15	+15/−15
Horizon field of view [°]	360	360
Vertical angular resolution [°]	-	2 ^1^
Horizon angular resolution [°]	-	0.4 ^1^
Rotation rate [Hz]	10	10
Points per second	144,000 ^2^	144,000 ^2^

^1^ Parameter cannot be set in simulation but depends on other parameters. ^2^ Parameter is not explicitly defined for VLP-16 lidar but can be computed out of other parameters, according to Equation (7).
